# Formulation, Quality Control and Stability Study of Pediatric Oral Dextrose Gel

**DOI:** 10.3390/ph18020204

**Published:** 2025-02-03

**Authors:** Edouard Lamy, Caroline Orneto, Oumil Her Abdou Ali, Lyna Kireche, Fanny Mathias, Cyrielle Bouguergour, Florence Peyron, Nicolas Primas, Christophe Sauzet, Philippe Piccerelle, Anne-Marie Maillotte, Veronique Brevaut-Malaty, Pascal Rathelot, Patrice Vanelle, Christophe Curti

**Affiliations:** 1 Service Central de la Qualité et de L’information Pharmaceutiques (SCQIP), Pharmacy Department, Assistance Publique-Hôpitaux de Marseille, 13005 Marseille, France; oumiabdouali@gmail.com (O.H.A.A.); lyna.kireche@gmail.com (L.K.); nicolas.primas@ap-hm.fr (N.P.); pascal.rathelot@ap-hm.fr (P.R.); patrice.vanelle@ap-hm.fr (P.V.); christophe.curti@ap-hm.fr (C.C.); 2 UMR 7287 CNRS, Institut des Sciences du Mouvement ISM, Faculté des Sciences du Sport, Aix-Marseille University, 13288 Marseille, France; 3 CNRS, IRD, IMBE, Avignon University, Aix Marseille University, 13397 Marseille, France; caroline.orneto@univ-amu.fr (C.O.); christophe.sauzet@univ-amu.fr (C.S.); philippe.piccerelle@univ-amu.fr (P.P.); 4 CNRS, Institut de Chimie Radicalaire ICR, UMR 7273, Equipe de Pharmaco-Chimie Radicalaire, Aix-Marseille University, 13015 Marseille, France; fanny.mathias@ap-hm.fr; 5 Pharmacy Department, Marseille Nord University Hospital Pharmacy, Assistance Publique-Hôpitaux de Marseille, 13015 Marseille, France; cyrielle.bouguergour@ap-hm.fr (C.B.); florence.peyron@ap-hm.fr (F.P.); 6 Department of Neonatology, Larchet University Hospital, 06200 Nice, France; maillotte.am@chu-nice.fr; 7 Department of Neonatology, Marseille Nord University Hospital, Assistance Publique-Hôpitaux de Marseille, 13015 Marseille, France; veronique.brevaut@ap-hm.fr

**Keywords:** dextrose, compounding, stability, diffusion, HPLC, rheology

## Abstract

**Background/Objective:** Little information is available on the stability and quality controls of compounded 40% dextrose gel required to ensure its safe use in the treatment and prevention of neonatal hypoglycemia. Whether its efficacy relies on buccal absorption also remains uncertain. This study investigates the stability, microbiological safety, rheological properties and dextrose diffusion of a compounded 40% oral dextrose gel, ensuring it can be widely compounded and stored for clinical use. **Methods:** A 40% dextrose gel compounded with anhydrous dextrose, carboxymethylcellulose, citric acid, sorbic acid and sterile water was subjected to quality control measures including a dextrose content assay, degradation product analysis, microbiological testing and preservative efficacy. Stability studies were conducted at refrigerated (4–8 °C) and ambient temperatures for 7 days and 3 months, respectively. Rheological properties were assessed, and dextrose permeation was measured through an artificial membrane model that mimics a biological membrane. **Results:** The compounded gel demonstrated stability for up to 7 days at ambient temperature and 90 days when refrigerated. The dextrose content remained within the acceptable range (90–110%) and microbiological tests confirmed compliance with safety standards. The gel exhibited the consistent rheological properties and shear-thinning behavior appropriate for oral mucosal administration. In vitro permeation studies showed no evidence of dextrose diffusion with a long lag time followed by a low steady-state permeation flux. **Conclusions:** This study validates the compounding process of a stable 40% oral dextrose gel formulation for neonatal hypoglycemia management, which meets quality control criteria and can be safely administered in clinical practice, offering a cost-effective and safe alternative for neonatal care.

## 1. Introduction

Although the definition of clinically significant neonatal hypoglycaemia remains controversial [1,2], it is the most common metabolic condition in the immediate postnatal period, affecting up to 15% of all babies and up to 50% of at-risk newborns [3,4]. While neonatal hypoglycemia is a global concern, evidence for neonatal hypoglycemia prevalence in low- and lower-middle-income countries is scarce [5]. The prevalence of neonatal hypoglycemia is likely underestimated in these countries because of low healthcare resources [6] and is also likely higher than in high-income countries because of the higher prevalence of risk factors which predispose babies to it [5]. Common treatment for neonatal hypoglycemia includes more feeding with early breastfeeding and/or formula milk, and intravenous dextrose in neonatal intensive care if glycemia is not improved [7]. It is noteworthy that the lack of standardized definitions for neonatal hypoglycemia and inconsistent protocols for screening and treatment contribute to variability in treatment rates and outcomes [8]. Also, while millions of babies are treated for neonatal hypoglycemia in developed countries thanks to high resource settings, a lower and limited access to treatment, due to lower resources, is available for babies in low- and lower-middle-income countries [5].

In pediatric practice, oral dextrose gel can be prescribed to prevent or cure hypoglycemia in neonates. Based on several clinical studies, a recent meta-analysis concluded that the 40% oral dextrose gel used to treat hypoglycemia in newborn infants is probably useful to increase the correction of hypoglycemic events, to reduce the risk of major neurological disability at two years of age or older, to reduce the incidence of separation from the mother and to increase the likelihood of exclusive breastfeeding after discharge [9]. This treatment consists of a single-dose application of 0.5 mL/kg (200 mg/kg) 40% dextrose gel to the buccal mucosa of babies and is followed by breastfeeding. Dextrose gel administration could be repeated up to six times if hypoglycemia recurs within 48 h of life [10]. Moreover, as no adverse events were reported, the authors concluded that oral dextrose gel is probably an effective and safe first-line treatment for infants with neonatal hypoglycemia.

However, another recent meta-analysis concluded that 40% oral dextrose gel used not to treat but to prevent hypoglycemia probably reduces the risk of neonatal hypoglycemia in at-risk infants with exclusive breast milk feeding and probably reduces the risk of treatment for hypoglycemia without adverse effects [11]. Indeed, in view of its limited short-term benefits, the authors do not recommend prophylactic oral dextrose gel as a routine practice until additional information is available about the balance of risks and harms for later neurological disability. A recent meta-analysis also concluded that there is an absence of the superiority of oral dextrose gel versus a placebo in the prevention of neonatal hypoglycemia [12]. However, the small sample size of each study is a major limitation to such meta-analyses. Moreover, considering the low cost and the relative safety of oral dextrose gel, the authors did not advise against its use in the prevention of neonatal hypoglycemia. A recent randomized dosage trial investigated the effect of different doses of prophylactic dextrose gel on neurocognitive function and health at 6–7 years. The effect on the risk of combined neurocognitive impairment at 6–7 years of corrected age was not statistically significant but some long-term motor and cognitive benefits have been mentioned and need to be confirmed by school-age follow-up studies [13].

These data encouraged our pediatricians to prescribe 40% oral dextrose gel as a treatment but also to prevent neonatal hypoglycemia. Some available commercial preparations contain preservatives like parabens as well as flavor additives that are not adapted to pediatric healthcare [14]. In addition, in some commercial preparations, dextrose was not found to be uniformly distributed within tubes, which may yield variable results in terms of its efficacy for neonatal hypoglycemia [14,15]. Moreover, although compounding was falsely considered to be a past practice until recently, new clinical practices, drug shortages and the need for different alternatives have highlighted the interest in this compounding practice. These findings, as well as the low costs of compounding [16], encouraged our hospital pharmacy to produce 40% oral dextrose gel to manage neonatal hypoglycemia inexpensively.

However, in the literature, the formulation of compounded 40% dextrose has rarely been specified and with no evidence of all of the specified quality controls being performed in compliance with European and/or United States Pharmacopeia (USP) requirements to validate the compounding process and secure the delivery [17,18]. Also, in a previous study by Rivano et al., the 30-day stability under refrigerated conditions (2–8 °C) of 40% dextrose gel, prepared from a 50% commercial glucose solution, carboxymethyl cellulose, glycerol, sodium propyl paraben and methyl paraben as preservatives, was determined [18]. However, important quality controls were lacking in the study to validate pharmaceutical compounding and stability, with no determination of dextrose content as well as no determination of preservative efficacy and of the specified microorganisms for oral and buccal administration.

Therefore, the main objective of our work was to develop a stable 40% oral dextrose gel that meets the requirements of our hospital’s pediatricians to administer it to neonates and which can be compounded easily and stored to be dispensed widely. To this end, we compounded a 40% oral dextrose gel without paraben, but with citric and sorbic acids as preservatives. We performed the required quality controls (dextrose content, degradation product evaluation, including the most toxic, 5-Hydroxymethylfurfural (5-HMF), pH measure and microbiological count), but also specified microorganism research, including *Escherichia coli*, *Staphylococcus aureus* and *Pseudomonas aeruginosa*, and preservative efficacy, to secure its delivery. We also determined the stability of homogeneous batches of dextrose gel stored either at 4 °C for 7 days or at room temperature for 4 months, including rheological parameters.

While trials using dextrose gel highlighted that the efficacy of dextrose gel may lay in dextrose absorption from the buccal mucosa rather than gastrointestinal absorption, this remains unclear according to the sparse results available [19,20,21]. To this end, we also investigated dextrose absorption from the compounded buccal gel through an artificial membrane described in the literature as a good model for buccal absorption [22].

## 2. Results

### 2.1. Dextrose Gel Formulation

For the compounding of a batch of 20 syringes (5 mL) filled with 40% (*w*/*v*) dextrose gel, 40.0 g of anhydrous dextrose (Cooper, Melun, France), 2.0 g of carboxymethylcellulose sodium salt (99.5%/225.1536, Caesar, & Loretz GmbH, Hilden, Germany), 0.05 g of sorbic acid (Inresa Pharmaceutical, Bartheim, France) and 0.07 g of citric acid (Caesar, & Loretz GmbH, Hilden, Germany) were weighted on a precision balance (XT 120A, Precisa Instrument Dietikon, Switzerland) and poured into a mortar. The powders were gently mixed with a pestle for 5 min. Sterile water (73.4 g) was weighed on a precision balance and added in fractions to the powder. After the addition of all of the sterile water, the mixture was left for 15 min and then mixed again for 2 min every 15 min for one hour. The mixture was left for 2–2.5 h until the gel became homogeneous. The gel was then packaged into 5 mL polypropylen syringes (Enfit^TM^, Vigon, Ecouen, France) with a cannula (Enfit^TM^, Vigon, Ecouen, France) and sealed with suitable caps (Enfit^TM^, Vigon, Ecouen, France).

### 2.2. Method Validation and Quality Control

#### 2.2.1. Dextrose

The dextrose content assay using spectrophotometric method was proven to be linear between 0.4 and 4 mg/mL and validated at the three concentration levels studied (1.44, 1.60 and 1.76 mg/mL). These concentrations were selected from the theoretical dextrose content of diluted samples (1.60 mg/mL) and the normal values ensuring sample conformity (90–110% of the theoretical dextrose content). The results of the method validation are summarized in Table 1.

#### 2.2.2. 5-HMF

5-HMF is a relevant degradation product of dextrose with plausible developmental toxicity when administrated orally as explained in the discussion of this work. To our knowledge, there is no specified limit value for 5-HMF content in compounds orally administered to pediatric patients and the sole reference is in the USP monograph “*Dextrose Injection*” [23]. Even though the toxicity of parenterally administered drugs is often higher than orally administered ones, we had to select this unique value in the absence of any other reference. However, in the USP monograph “*Dextrose Injection*”, the concentration limit values for 5-HMF and related substances are not accurately quantified since the acceptance criteria are defined as an absorbance lower than 0.25 at 284 nm for a 4 mg/mL dextrose solution. Therefore, to correlate this absorbance value to 5-HMF concentration, we prepared 5-HMF standard solutions for calibration curves that were analyzed spectrophotometrically with the USP protocol. An absorbance value of 0.25 was found for a 5-HMF standard solution of 15 µmol/L. This limit value transposed to a 40% dextrose gel corresponds to a 5-HMF concentration (M = 126.11 g/mol) lower than 1.89 µg/mL. However, the dextrose raw material monograph in the USP does not evaluate 5-HMF content, but a maximal content lower than 0.10% is specified for an unspecified impurity [23]. This limit value transposed to a 40% (400 mg/mL) dextrose gel corresponds to a content of unspecified impurity (including 5-HMF) not higher than 400 µg/mL. However, regarding a dextrose gel dosage regimen, for a 5-HMF potent toxicity and for high-risk neonate patients, a 50 µg/mL maximal value for 5-HMF was chosen for the stability study of 40% dextrose gel administered orally. This maximal value is explained in the discussion of the present work.

The 5-HMF dosing method described in the USP for the quality control of a dextrose solution in water is a non-separative method. A separative method validated as stability-indicating was needed to determine the appearance and evolution of 5-HMF during a stability study performed on a complex dextrose mixture. Therefore, a high-performance liquid chromatography with a UV diode array detector (HPLC-UV-DAD) method on an HPLC apparatus (Dionex Ultimate 3000, Dionex Softron GmbH, Germering, Germany) with pre-column derivatization was validated. Data analysis was performed using Chromeleon^®^ software (Version 7.2.8, Thermo Fisher Scientific, Waltham, MA, USA). The 5-HMF content assay by HPLC-UV-DAD was linear between 2.5 and 50.0 µmol/L (3.15–63.05 µg/mL in undiluted dextrose gel samples) and was validated at the three studied concentration levels (10, 15 and 20 µmol/L) as reported in Table 2. The limit of quantification (LOQ) and limit of detection (LOD) of 5-HMF in dextrose gel were not precisely determined and were estimated to be equal to the lowest level of linearity (3.15 μg/mL).

### 2.3. Forced Degradation Studies

Table 3 presents the results of the forced degradation study. For each degradation condition, samples were analyzed at several time points to approximate a 20% decrease in dextrose content (determined with the spectrophotometric method). When 20% degradation was reached, the samples were also analyzed by HPLC-UV-DAD to determine the 5-HMF content and identify other unidentified impurities/degradation products.

Our results show that tandem analysis, consisting of a non-separative method able to quantify dextrose content and a separative method able to detect impurities and to quantify the main degradation product (5-HMF), can be considered as stability-indicating.

### 2.4. Microbiological Analyses and Preservative Efficacy

TAMC and TYMC were validated by the surface spread method. In pediatric practice, 40% (*w*/*v*) dextrose gel is orally administered. For its administration, the gel is spread on the oral mucosa, but a variable quantity is also swallowed. Therefore, specified microorganisms for both oral administration (*Escherichia coli*) and buccal administration (*Staphylococcus aureus* and *Pseudomonas aeruginosa*) were controlled according to European Pharmacopeia specifications [24]. The suitability of these three methods was validated. Following the microbiological method’s validation, experimental batches of compounded dextrose gel were analyzed. On the day when batches of the dextrose gel were compounded (D0), TAMC was lower than 200 colony forming units per milliliter (CFU/mL) of dextrose gel, TYMC was lower than 20 CFU/mL and none of the specified microorganisms were detectable.

The preservative efficacy of the dextrose gel was also tested. Our first tested formula contained only citric acid (0.07% *w*/*v*) used as a preservative, but the test for preservative efficacy failed. *Escherichia coli* and *Staphylococcus aureus* growth was inhibited with a decrease in bacterial count not lower than 3 log after 14 days but also after 28 days. However, *Candida albicans* and *Aspergillus brasiliensis* growth was not inhibited at all. We therefore added sorbic acid (0.05% *w*/*v*) to the citric acid (0.07% *w*/*v*), and the resulting formula had an efficient preservative effect, as *Aspergillus brasiliensis* and *Candida albicans* growth was also inhibited both after 14 days and 28 days, with a count decrease higher than 1 log.

### 2.5. Content and Content Uniformity

The control of dextrose content in syringes filled with dextrose gel and of dextrose content uniformity was adapted from European Pharmacopoeia recommendations, regarding the small size of batches produced [25]. In total, 20 syringes per batch were produced as a single-dose nonsterile preparation with a 40% (*w*/*v*) dextrose content. Theoretically, such a single-dose drug with a content higher than 25% must be controlled for its uniformity with a 2.9.40. test (mass variation). This analysis implies the quantification of one sample and the destructive weight of ten samples (the weighing of a “syringe + dextrose gel” sample followed by weighing of an “empty syringe” allow for the calculation of the weight of dextrose gel for each sample), followed by the calculation of an acceptance value which must be lower than 15% to ensure batch uniformity. In a batch of 20 syringes, 10 syringes cannot be destructively weighed. Therefore, just before the batch production, the qualified personnel (a pharmacy technician or hospital pharmacist) weighed ten syringes without emptying them and sent the weighing ticket and two syringes. One syringe was analyzed for microbiological conformity (TAMC, TYMC and the absence of *Escherichia coli*, *Staphylococcus aureus* and *Pseudomonas aeruginosa*), and the second one was analyzed for dextrose content using the spectrophotometric method.

The dextrose content and weights of ten syringes filled with dextrose gel allowed for the determination of an acceptance value in a similar way to that described in the European Pharmacopoeia (2.9.40; mass variation). The non-destructive weighing of syringes is a bias, as the weight variability of the empty syringes is not considered. However, such adaptations from the European Pharmacopeia recommendations were needed, given the small size of the controlled batches. The small size of the batches imposed on us the need to reduce the number of samples dedicated to the control, but the number of syringes per batch could be increased if dextrose gel became widely prescribed. If the number of syringes per batch allows, a mass variation test will have to be performed to control batch uniformity.

### 2.6. Rheological Parameters

The peak-hold flow mode involves applying a shear rate of 100 s^−1^ for 180 s to ensure the determination of a stable viscosity value for compounded dextrose gel. The absence of any variation in the viscosity value during the 180 s of the test has been verified to validate the viscosity value obtained for each tested dextrose gel. This test was performed to assess whether thixotropic behavior was present and to check the steady state. The chosen gradient represents the stress undergone by a product in the oral cavity during its spreading and swallowing. On the day when batches of dextrose gel were compounded (D0), a viscosity value of 481.5 ± 19.3 mPa.s was determined (Table 4).

**Figure 1:**
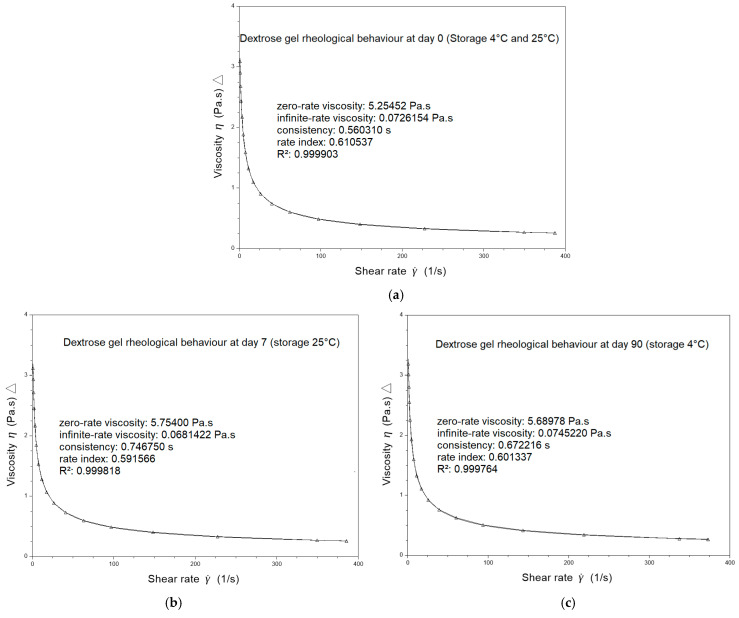
The rheological behavior of 40% dextrose gel as a function of shear rate: (**a**) on the day the dextrose gels were compounded (D0); (**b**) after 7 days of storage under ambient conditions (25 °C/60% RH); (**c**) after 90 days of storage under refrigerated conditions (2–8 °C).

The rheological behavior of dextrose gels was also investigated using the flow sweep mode by applying different shear stresses to achieve the corresponding shear rate values. A representative flow curve obtained for dextrose gel on the day the batches were compounded (D0) exhibits the shear-thinning behavior of the gel with a rate index of 0.61, determined with the CROSS model (Figure 1a).

### 2.7. Stability Study

**Figure 2:**
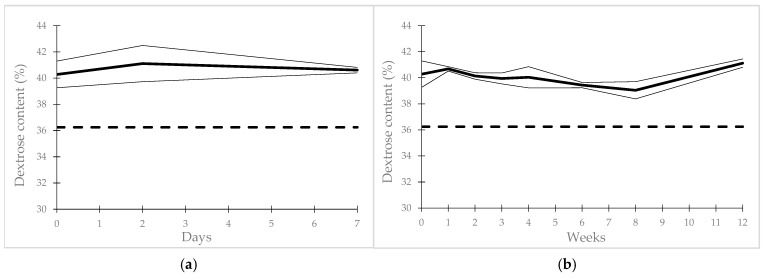
Stability of dextrose content in gel: (**a**) under ambient conditions (25 °C/60% RH); (**b**) under refrigerated conditions (2–8 °C). Bold lines: mean dextrose gel content; thick lines: 95% confidence interval; dashed lines: 90 percent of theoretical initial dextrose gel content.

The 40% (*w*/*v*) dextrose gel in syringes stored at 25 °C/60% room humidity (25 °C/60% RH) remained stable for 7 days, and for 90 days when stored at 2–8 °C. In both storage conditions, the dextrose content remained between 90% and 110% of its initial value (Figure 2 and Table 4 and Table 5) without any appearance of 5-HMF (Table 4 and Table 5). Moreover, pH did not significantly vary through the study, whatever the conditions (Table 4 and Table 5). At the all-time point of stability, tested for in dextrose gel kept in ambient or refrigerated conditions, the microbiological results were also compliant with European Pharmacopeia specifications for microbiological contamination with TAMC and TYMC, both of which remained lower than the method detection limit (<10 CFU/mL), and the specified microorganisms that remained were not detectable (Table 4 and Table 5). Also, the preservative efficacy of the compounded dextrose gels remained effective for 7 days when the dextrose gels were stored at ambient temperature and for 90 days when they were stored at 2–8 °C (Table 4 and Table 5).

Viscosity did not vary for dextrose gel stored for 90 days at 2–8 °C (Table 4) nor for dextrose gel stored for 7 days at 25 °C/60% RH (Table 5). A small variation in the process factor or in the quality of raw materials can have an influence on the value of the rheological parameters. A tolerance of 10% is widely accepted in the variability of microstructure parameters in the characterization of topical semi-solids [26]. The representative flow curves obtained for dextrose gel after 7 days of storage at 25 °C/60% RH (Figure 1b) and after 90 days of storage at 2–8 °C (Figure 1c) exhibit the remaining shear-thinning behavior of the gel with respective rate indexes of 0.59 and 0.60 determined with the CROSS model.

### 2.8. Dextrose Permeation Through Artificial Membrane

**Figure 3:**
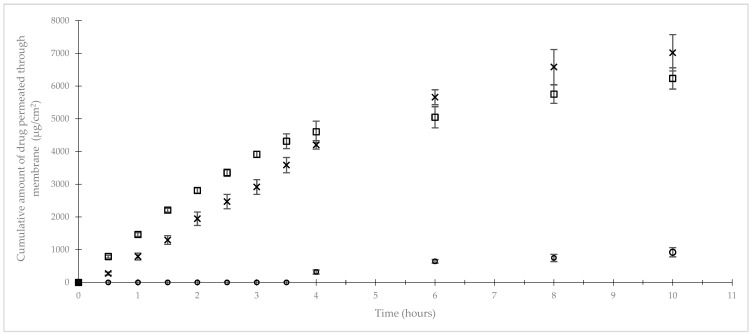
In vitro permeation profile through artificial membrane of different drugs. (○) Dextrose; (□) paracetamol; (×) caffeine.

The cumulative amount of dextrose that permeated through a membrane that mimics a biological membrane is provided in Figure 3 and Table 6. The permeation profiles of dextrose, as well as paracetamol and caffeine used as positive controls, through an artificial membrane impregnated with lipids were obtained by plotting the cumulative amount of these molecules that permeated per unit area versus time. The results are reported in Figure 3 and Table 6. The release profile of dextrose showed a long lag time of 2.84 h ± 0.19 h followed by a low steady-state permeation flux of 163.24 ± 27.91 µg/cm^2^/s (R^2^ = 0.98) that was determined from the slope of the linear portion (4–10 h) of the curve. When compared to dextrose, the release profiles of both paracetamol and caffeine showed shorter lag times (0.10 ± 0.04 h and 0.46 ± 0.19 h, respectively) and higher steady-state permeation fluxes (1670.88 ± 55.59 µg/cm^2^/s; R^2^ = 0.98 and 1234.81 ± 67.35 µg/cm^2^/s R^2^ = 0.98, respectively) that were both also calculated from the slope of the linear portion (0.5–3 h and 0.5–4 h, respectively) of their curves.

## 3. Discussion

In this work, two complementary methods were validated: the spectrophotometric quantification of dextrose content and the HPLC-UV-DAD quantification of 5-HMF/research on other dextrose impurities. These two methods result in a stability-indicating protocol able to evaluate dextrose gel stability.

During method validation, forced degradation experiments identified an increase in 5-HMF under heat and oxidative conditions. This observation agrees with the conclusion of a study on factors generating dextrose degradation products in sterile dextrose solutions for infusion. The authors reported that the temperature and duration of the sterilization of the dextrose solutions and the oxygen permeability of the container have a significant influence on the increase in 5-HMF [27].

In the literature, there are contradictory findings on the possible carcinogenicity of 5-HMF [28]. Experimental studies performed on animals with high doses of 5-HMF orally administered suggest toxicities on several organs, but also a developmental toxicity [29]. As a product of the Maillard reaction, 5-HMF can be found in many foods with estimated ranges between 4 and 30 mg per person per day. The maximum 5-HMF exposure (from sources other than dried plums or caramel colors which have very high 5-HMF levels) is suggested to be lower than 500 µg/kg per day.

The USP monograph “*Dextrose*”, for raw materials used both for oral and parenteral medications, proposes a maximal content for unspecified impurities of 0.1%, which corresponds to 400 µg/mL of 5-HMF if applied to 40% dextrose gel. The USP monograph “*Dextrose Injections*” defines a maximal content of 1.89 µg/mL for 5-HMF but only for parenteral administration. In the present study, to evaluate oral dextrose gel stability, we defined a maximal 5-HMF value of 50 µg/mL, lower than those reported for dextrose as a raw material. Dextrose gel will be orally administered to high-risk patients (neonates) at a maximal dosage regimen of 0.5 mL/kg every 4 h [30]. With a 50 µg/mL maximal value for 5-HMF, the maximum exposure will be restricted to 200 µg/kg, more than twice lower than the maximum 5-HMF recommended exposure of 500 µg/kg found in the literature [23].

Then, 40% (*w*/*v*) dextrose gel was compounded and checked for its conformity to validate the compounding formulation and process. Dextrose gel was formulated with carboxymethylcellulose, as previously described in the literature [18]. However, it could be interesting in future works to study other hydrogel-forming reagents such as sodium alginate [31,32]. Such variation could allow for the comparison of critical parameters such as uniformity, dextrose diffusion and rheological parameters of compounded 40% dextrose gel. For the tested batches, syringes were filled with dextrose gel that contained not less than 90% and not more than 110% of the labeled amount of dextrose. Moreover, the content uniformity results were also compliant with the European Pharmacopeia standard, since the acceptance values were lower than 15%.

Regarding microbiological conservation, the first batch produced contained only one preservative, 0.07% (*w*/*v*) of citric acid and was not compliant with the test on preservative efficacy for fungal strains. A second batch was compounded, which contained a mixture of two preservatives, 0.07% (*w*/*v*) of citric acid and 0.05% (*w*/*v*) of sorbic acid. The test for preservative efficacy was compliant for both strains. This formula was therefore chosen and resulted in a final content per syringe (5 mL) of 3.5 mg of citric acid and 5.0 mg of sorbic acid. Sorbic acid and citric acid are commonly used as food additives. As a food additive, the acceptable daily intake (ADI) for sorbic acid was established as 11 mg of sorbic acid/kg bw per day [33]. The ADI for citric acid was not limited as this molecule is safe for consumers [34]. However, the nature and the quantity of the used preservatives in 40% (*w*/*v*) dextrose gel appear to be safe in pediatric practice.

Although the rheological behavior of compounded 40% (*w*/*v*) dextrose gel is not a mandatory parameter to validate the compounding process and secure the delivery, we considered its characterization important given its buccal administration consisted of spreading it against a neonate cheek. To our knowledge, there are no existing data for compounded dextrose gel in the literature. Nevertheless, the shear-thinning behavior of the selected gels was clearly demonstrated. This type of behavior allows the product to spread out in the baby’s mouth, making swallowing easier. In fact, the gel’s viscosity decreases significantly as the baby swallows.

The stability of 40% (*w*/*v*) dextrose gel was evaluated under two storage conditions. Compounded dextrose gel syringes were stable for 7 days when stored at 25 °C and 60% RH, and were stable for 90 days when stored at 2–8 °C. A previous work had already evidenced a 30-day stability for 40% dextrose gel when stored both at ambient temperature and between 2 °C and 8 °C [18]. However, in this study, the dextrose content, degradation products as well as rheological parameters were not determined. Moreover, the dextrose gel formulation contained sodium propyl paraben and methyl paraben that are not adapted to pediatric practice. The results of our stability study evidence that a formulation of 40% dextrose gel containing acceptable preservative amounts is stable for up to 3 months when stored at 2–8 °C. Such a stability duration could allow the compounding of larger batches of dextrose gel syringes in the future, enabling the rationalization of production time and volume and a resulting reduction in hospital costs.

The 40% (*w*/*v*) dextrose gel is efficient in treating hypoglycemia in newborn infants [9] and can also be used to prevent hypoglycemia in these patients [12]. How dextrose is contained in gel-rich blood circulation remains unclear given the contradictory results from sparse studies previously described as having low levels of evidence [19,20,21]. In our work, the in vitro permeation experiment did not evidence efficient dextrose diffusion through an artificial membrane, whereas caffeine and paracetamol showed good diffusion when compounded within the same gel formula. To our knowledge, this is the first work that determines and exhibits values for the permeation parameters of dextrose gel using an artificial membrane. Permeation parameters measured for paracetamol and caffeine are in accordance with those previously described, confirming the efficiency of this in vitro model in predicting dextrose’s buccal absorption [35]. It is noteworthy that the dextrose in the gel did not permeate through an artificial membrane that mimics a biological membrane in the first 3 h after gel spreading. Therefore, this observation supports the effectiveness of dextrose gel in the treatment of hypoglycemia in newborn infants due to the digestive absorption of dextrose contained in a gel, and consecutive to the swallowing of the dose applied on buccal mucosa, rather than the buccal diffusion of the dextrose. Despite these in vitro results, dextrose gel remains useful for the treatment of hypoglycemia in newborn infants, as it has been described as being inexpensive and less invasive than other forms of dextrose administration, and it does not disrupt mother–infant bonding [36].

## 4. Materials and Methods

### 4.1. Dextrose Content

Dextrose was quantified by spectrophotometry using an enzymatic method. Quantification was performed with the dextrose pharmaceutical secondary standard (Sigma-Aldrich Chemie GmbH, Taufkirchen, Germany), the content of which was previously checked with the standard reference material (Sigma-Aldrich Chemie GmbH, Taufkirchen, Germany) with the same procedure.

Theoretically, 40% oral dextrose gel contains 40% (*w*/*v*) of dextrose. One mL of dextrose gel was diluted with ultrapure water sq. 250 mL to achieve a theoretical dextrose content of 1.6 mg/mL. In a hemolysis tube, 4 mL of extemporaneously prepared glucose oxidase (GOD) reactant (Biolabo S.A.S, Mazy, France) and 40 µL of diluted dextrose gel (with a theoretical dextrose content of 1.60 mg/mL) were mixed. This mixture was vortexed for 1 min, incubated at ambient temperature for 20 min and transferred into a spectrophotometric tank. Absorbance was determined with an UV–vis spectrophotometer (UV MINI-1240, Shimadzu^®^, Kioto, Japan) at 500 nm against a blank (4 mL of reactant and 40 µL of ultrapure water). The measures were performed extemporaneously as the coloration remains stable for only 15 min, and slowly decreases.

With a precision balance (Cubis MCA 125P, Sartorius Lab Instruments GmbH, Gottingen, Germany), 80 mg of dextrose standard were weighed and solubilized with ultrapure water sq. 50 mL. The resulting solution (with a dextrose content of 1.60 mg/mL) was prepared twice by two independent technicians. One solution was analyzed in a single experiment, and the second was analyzed 6 times to evaluate system suitability (the RSD must be lower than 2%). Absorbance was measured with the same protocol as previously described for dextrose gel samples. The dextrose content in the samples was calculated from the comparison of the sample absorbance (SA) and mean absorbance values of the two standards (MA) with a coefficient that takes into account the dilutions. Dextrose content (in g/100 mL) = (SA/MA) × 40.0.

Method validation was performed as follows: Linearity was determined with seven concentrations ranging from 0.4 to 4.0 mg/mL (0.4, 0.8, 1.2, 1.6, 2.0, 3.0 and 4.0) prepared in quintuplicate from dextrose secondary standard dissolved in ultrapure water. Repeatability was determined from within-day variation measurements (n = 18), whereas intermediate precision and accuracy were determined from between-day variation measurements (n = 18). Three concentration levels were studied (1.44, 1.60 and 1.76 mg/mL)

### 4.2. 5-HydroxyMethylFurfural (HMF) Content

5-HMF content was determined by HPLC-UV-DAD after pre-column derivatization as previously reported [37]. The chromatographic method used an automatic HPLC-UV-DAD system with a 5 µm F5 100 A, LC column 100 × 4.6 mm (Kinetex, Phenomenex, California, USA). The mobile phase A consisted of an aqueous solution of ammonium formate (0.315 g sq. 1 L of ultrapure water) adjusted to pH 3.3 with glacial acetic acid. It was filtered through a Millipore 0.45 µm cellulose. The mobile phase B was methanol. The gradient mode at a flow of 0.4 mL/min was used with the following parameters: (% of mobile phase A) T 0–1 min 80%; 2.5–12 min 69%; 13–18 min 63%; 22 min 45%; 22.2–25 min 10%; and 25.1–30 min 80%. The wavelength for derivatized 5-HMF detection was 335 nm, the injection volumes were 10 µL and the column temperature was 50 °C.

Method validation, system suitability tests (SSTs) and quality controls were performed with a 15 µmol/L (1.89 µg/mL) solution of 5-HMF standard (Sigma-Aldrich Chemie GmbH, Taufkirchen, Germany) in ultrapure water. This standard solution was derivatized with the same protocol as the samples.

The derivatization reactant was made from 5 mL of a 1 M solution of N-2-Hydroxyethylpiperazine-N′-2-ethanesulfonic acid (Thermo Fisher Scientific, New York, NY, USA) in which 200 mg of Ortho-Phenylenediamine (Sigma-Aldrich Chemie GmbH, Taufkirchen, Germany) was solubilized.

For derivatization, 180 µL of the sample (1:10 dextrose gel dilution in ultrapure water or 5-HMF standard solution) was mixed with 20 µL of derivatization reactant. The mixture was kept for 4 h at ambient temperature (25 °C, 60% RH) and protected from light before analysis.

Method validation was performed as follows: Linearity was determined with seven concentrations ranging from 2.5 to 50 µmol/L (2.5, 5, 10, 20, 30, 40 and 50) prepared in quintuplicate from 5-HMF standard dissolved in ultrapure water and derivatized. Repeatability was determined from within-day variation measurements (n = 18), whereas intermediate precision and accuracy were determined from between-day variation measurements (n = 18). Three concentration levels were studied (10, 15 and 20 µmol/L).

After method validation, system suitability tests (SSTs) were performed routinely with a 5-HMF standard solution analyzed six times. Conformity criteria were established as follows: capacity factor > 2; derivatized 5-HMF resolution > 2; number of theoretical plates > 2000; tailing factor ≤ 2 and relative standard deviation of the six analyses < 2%.

### 4.3. Forced Degradation Studies

Samples of 40% oral dextrose gel were exposed to several stress conditions including acidity (HCl 1 N final concentration 0.5 N), basicity (NaOH 1 M final concentration 0.5 M), heat (80 °C in a sealed tube), light (sunlamp) and oxidation (KMnO_4_ 20 mg/mL final concentration). Each sample was analyzed using the spectrophotometric method for dextrose content and by the HPLC-UV-DAD method for 5-HMF content. In our protocol, dextrose oxidation was studied with KMnO_4_ instead of H_2_O_2_ to avoid any interference with the GOD reactant used to determine dextrose content.

### 4.4. Microbiological Analyses

Microbiological analyses of 40% oral dextrose gel were validated according to European Pharmacopoeia with five reference strains (Bioball^®^ or Microbiologics^®^, Biomerieux SA, Sydney, Australia): *Staphylococcus aureus* ATCC6538, *Bacillus subtilis* NCTC10400 *Pseudomonas aeruginosa* ATCC9027, *Candida albicans* NCPF3179 and *Aspergillus braziliensis* NCPF2275. The microbiological analyses were carried out in Tryptic Soy Agar (TSA) (Beckton Dickinson GmbH, Heidelberg, Germany) and Sabouraud chloramphenicol gentamicin agar (Biomerieux SA, Marcy l’Etoile, France) under a Microbiological Safety Cabinet (MSC) (Herasafe^®^ KS, Thermo Scientific, Langenselbold, Germany). Also used were 9 mL tubes of pharmacopeia diluent with a neutralizer (VWR chemicals, Leuven, Belgium) and incubators (Heratherm^®^ and Heraeus^®^, Thermo Scientific, Langenselbold, Germany). Microbiological analyses of dextrose gel were carried out under a microbiological safety cabinet using a surface-spread method. The suitability of the method was proved according to European Pharmacopeia: five reference strains (*Staphylococcus aureus*, *Bacillus subtilis*, *Pseudomonas aeruginosa*, *Candida albicans* and *Aspergillus braziliensis*) were suspended in pH 7.2 sterile phosphate buffer to obtain 100–1000 CFU/mL. Then, 100 µL (10–100 CFU) of each strain suspension were aliquoted in five tubes containing 5 mL of pharmacopeia diluent (reference strains alone), in five tubes containing 4 mL of pharmacopeia diluent and 1 mL of dextrose gel. Two tubes were prepared as negative controls with 4 mL of pharmacopeia diluent/1 mL of dextrose gel. Each tube was vortexed for 2 min.

Four times, 500 µL was aliquoted from each tube to inoculate two TSAs and two Sabouraud agars. The TSAs were incubated for 3 days in an incubator at 30–35 °C and the Sabouraud agars for 5 days at 20–25 °C. At the end of the incubation, for each reference strain, the mean number of the CFU of “dextrose gel + reference strain” samples and the mean number of the CFU of “dextrose gel + reference strain” did not vary more than twice the mean number of the CFU of “reference strain alone” samples.

Following the microbiological method validation, TAMC and TYMC were determined in experimental batches of dextrose gel. An amount of 1 mL of dextrose gel was diluted in 4 mL of pharmacopeia diluent. The tube was then vortexed and two TSA and two Sabouraud agars were inoculated with 500 µL of the dilution. The mean number of CFU present on the TSA and on Sabouraud agar was determined and expressed as the number of UFC/mL of dextrose gel. For dextrose gel, TAMC had to be lower than 200 CFU/mL and TYMC lower than 20 CFU/mL, according to European Pharmacopeia specifications [16].

### 4.5. Specified Microorganisms

The *Escherichia coli* research was validated with two reference strains (Bioball^®^ or Microbiologics^®^, Biomerieux SA, Sydney, Australia), with *Escherichia coli* ATCC8739 as the positive control and *Staphylococcus aureus* ATCC6538 as the negative control. One mL of dextrose gel was solubilized in Tryptic Soy Broth (TSB) (Biomerieux SA, Marcy l’Etoile, France) and incubated at 35 °C for 24 h. The next day, under a microbiological safety cabinet, 1 mL of TSB was transferred in 100 mL of McConkey broth (Clearline, Bernolsheim, France) which was incubated at 45 °C for another 24 h. Finally, the next day, under a microbiological safety cabinet, 500 µL was aliquoted from the McConkey broth to inoculate McConkey agar (Beckton Dickinson GmbH, Heidelberg, Germany). The McConkey agar was incubated at 35 °C for 72 h.

The *Staphylococcus aureus* research was validated with two reference strains Bioball^®^ or Microbiologics^®^, Biomerieux SA, Sydney, Australia), with *Staphylococcus aureus* ATCC6538 as the positive control and *Escherichia coli* ATCC8739 as the negative control. One mL of dextrose gel was solubilized in TSB and incubated at 35 °C for 24 h. The next day, under a microbiological safety cabinet, 500 µL was aliquoted from TSB to inoculate Chapman agar (Beckton Dickinson GmbH, Heidelberg, Germany). The Chapman agar was incubated at 35 °C for 72 h.

The *Pseudomonas aeruginosa* research was validated with two reference strains (Bioball^®^ or Microbiologics^®^, Biomerieux SA, Sydney, Australia), with Pseudomonas aeruginosa ATCC9027 as the positive control and *Escherichia coli* ATCC8739 as the negative control. One mL of dextrose gel was solubilized in TSB and incubated at 35 °C for 24 h. The next day, under a microbiological safety cabinet, 500 µL was aliquoted from the TSB to inoculate the cetrimide agar. The cetrimide agar was incubated at 35 °C for 72 h.

### 4.6. Preservative Efficacy

Preservative efficacy was studied with four reference strains (Microbiologics^®^, Biomerieux SA, Sydney, Australia): *Staphylococcus aureus* ATCC6538, *Pseudomonas aeruginosa* ATCC9027, *Candida albicans* NCPF3179 and *Aspergillus braziliensis* NCPF2275. At D0, 4 dextrose gel samples were contaminated with 10^5^ to 10^6^ CFU/mL from each reference strain and one dextrose gel sample was used as a negative control. The evolution of the contamination was studied with TAMC and TYMC on the day of inoculation and 2 weeks and 4 weeks after inoculation on agars. The logarithmic decrease in the microorganism count must be at least equal to a factor of 3 for the bacterial strains and at least equal to a factor of 1 for the fungal strains after 2 weeks. After 4 weeks, these values cannot be higher than those reported at D14.

### 4.7. Rheological Parameters

All rheological measurements of gels were carried out on a rheometer (Discovery hybrid rheometer-2, TA Instrument^®^, New Castle, DE, USA) with an aluminum cone and plate (a 40 mm diameter cone with an angle of 2°; 53 µm gap) geometry. Data analysis was performed using TRIOS^®^ software (Version 5.1.1.46572, TA Instrument^®^, New Castle, DE, USA). The temperature was controlled at 25 °C using a Peltier plate and a solvent trap was used to minimize sample evaporation. Measurements on each sample were performed in triplicate. A peak hold measurement was applied at 100 s^−1^ for 180 s to determine the viscosity value. The rheological behavior of the dextrose gels was also determined using the flow sweep mode which consisted of applying a stress rate range of 3 to 100 Pa to the dextrose gel samples to determine the relationship between the shear stress and shear rate. All the measurements were performed in a steady-state mode. The flow curves obtained were modeled with the CROSS model to determine the rate index (behavior index).

### 4.8. Stability Study

Three batches of 40% (*w*/*w*) dextrose gel syringes were compounded and stored protected from light. The stability of the dextrose gel was investigated for 3 months under refrigerated conditions in a fridge (2–8 °C) with analyses at Day 0 (D0), Day 2 (D2), Day 7 (D7), Day 15 (D15), Day 21 (D21), Day 30 (D30), Day 45 (D45), Day 60 (D60) and Day 90 (D90). Stability was also investigated when dextrose bulk samples were stored under ambient conditions in a climatic chamber (25 °C/60% RH) with analyses at D0, D2 and D7. The dextrose content, degradation products, rheological parameters and pH were evaluated in triplicate at every time point and for each batch. TAMC, TYMC and specified microorganism analysis were performed only for one of these batches at every time point. Preservative efficacy was evaluated only at D0, D30, D60 and D90 when the samples were stored in a fridge (2–8 °C) and at D0 and D7 at ambient temperature. Significant changes in dextrose content were defined as a 10% change in dosage from its initial value as previously reported for hospital-compounded preparations [38,39,40,41].

### 4.9. In Vitro Permeation Experiment

Classical artificial membranes used for skin permeation studies are not suitable to study dextrose’s buccal absorption since they do not reflect the mucosal barrier. However, the efficacy of an in vitro procedure to predict drug buccal absorption using an artificial membrane impregnated with lipids has previously been described. Dextrose permeation was determined according to this previously reported procedure with minor modifications [22]. Briefly, a lipid mixture constituted of 4.7 g of 1-octanol (Sigma-Aldrich Chemie GmbH, Taufkirchen, Germany), 0.15 g of phosphatidylcholine (Sigma-Aldrich Chemie GmbH, Taufkirchen, Germany) and 0.15 g of cholesterol (Sigma-Aldrich Chemie GmbH, Taufkirchen, Germany) was prepared in a beaker (octanol/phosphatidylcholine/cholesterol ratio 94%/3%/3% *w*/*w*/*w*). The mixture was placed under magnetic stirring for 1 h. The cellulose acetate–nitrate mixture membrane (0.025 µm MCE membrane^®^, Sigma-Aldrich Chemie GmbH, Taufkirchen, Germany) was weighed and impregnated by submersion for 1 h in the lipid mixture. Impregnated membranes were then interposed between two absorbing papers to eliminate the excess lipids, and were weighed again to determine the percentage of lipid impregnation. The membranes were used on the day of the experiment to avoid membrane drying or lipid degradation.

The permeation of 40% (*w*/*v*) dextrose gel was studied with this artificial membrane. Although this artificial membrane’s efficacy had already been proven and compared with porcine buccal mucosa with naproxen [22], and since paracetamol and caffeine can be administered through buccal mucosa, positive permeation controls were performed with compounded 14% (*w*/*v*) paracetamol gel and 20% (*w*/*v*) caffeine gel using the same gel formula used for dextrose gel compounding.

An in vitro permeation study was conducted using a Phoenix RDS Automated Diffusion platform (model DB-6 Manuel, Teledyne Hanson Research, Chatsworth, USA) equipped with twelve in-line vertical diffusion cells enabling simultaneous in vitro release experiments. Each 15 mL cell receptor compartment, Franz cell, was filled with simulated plasma consisting of a phosphate-buffer solution (0.8 g/L Na_2_HPO_4_, 0.15 g/L KH_2_PO_4_ and 9 g/L NaCl) at pH 7.4 for the experiments with dextrose and caffeine gels and at pH 5.8 for the paracetamol to ensure good enough solubility to maintain sink condition. The simulated plasma was maintained at 32 ± 1 °C by the dry heat block and continuously stirred at 200 rpm. For all 3 gels, approximately 1.15 g (1 mL) was uniformly spread on the dosage chamber with an extemporaneously impregnated membrane placed between the dosage chamber and the simulated plasma. The cell was sealed by placing a cover on top of the dosage chamber. At given time intervals (0.5, 1, 1.5, 2, 2.5, 3, 3.5, 4, 6, 8 and 10 h), aliquots (0.5 mL) of simulated plasma were withdrawn from the cell receptor compartment through the sampling port and replaced by an equal volume of fresh pre-warmed simulated plasma, to keep the volume constant and sink conditions constant. The experiments were reproduced 8 times for the 3 different gels and the dilution effect was considered to determine the cumulative amounts of dextrose, caffeine and paracetamol. Their concentrations in each sample of withdrawn simulated plasma were determined.

The glucose contents were determined as previously described in this work. The direct determination of paracetamol content was performed at 243 nm using a UV–visible spectrophotometer (UV 2401PC, Shimadzu Scient. Inst., Colombia, SC, USA) according to a previously published method [42]. The caffeine contents were measured using the HPLC-UV method validated to be stability-indicating in our laboratory. Briefly, 10 µL of sample was injected into an automatic HPLC-UV-DAD apparatus (Dionex Ultimate 3000, Dionex Softron GmbH, Germering, Germany) with an RP18 column (1000 Å, 5 μm, 4 × 250 mm) (Lichrospher^®^, Sigma-Aldrich Chemie GmbH, Taufkirchen, Germany). Data analysis was performed using Chromeleon^®^ software (Version 7.2.8, Thermo Fisher Scientific, Waltham, USA). The mobile phase consisted of a mixture of ammonium acetate buffer adjusted to pH 4 (88%, *v*/*v*) and acetonitrile (12%, *v*/*v*), and the wavelength for caffeine detection was 274 nm. The permeated profiles of the gels were plotted as the cumulative amount of dextrose, paracetamol and caffeine diffused per unit area of membrane versus time. The flux (μg/cm^2^/h) and lag time (h) estimates were generated using the free Skin and Membrane Permeation Data Analysis (SAMPA) software (version 1.04) developed by Bezrouk et al. for skin and membrane permeation data analysis [43].

## 5. Conclusions

In the present work, we validated the formulation process and secured the delivery of batches of 20 syringes (5 mL) filled with 40% (*w*/*v*) dextrose gel compounded by the hospital pharmacy with anhydrous dextrose (40 g), carboxymethylcellulose sodium salt (2 g), sorbic acid (0.05 g), citric acid (0.07 g) and sterile water (74.5 g). This formula can be administered as a treatment for neonatal hypoglycemia. Its quality control implies the determination of batch uniformity (dextrose content and the weighing of ten samples) and research on its microbiological contamination (TAMC, TYMC, Absence of *Escherichia coli*, *Staphylococcus aureus* and *Pseudomonas aeruginosa*). Compounded dextrose gel syringes are stable for 7 days when stored at ambient temperature, and are stable for 90 days when stored in a fridge. These results allow for the compounding of batches of 20 syringes, or multiples of 20 if the forecast consumption justifies it, as well as of larger batches of dextrose gel syringes, enabling the rationalization of production time and volume and a resulting reduction in hospital costs.

## Figures and Tables

**Table 1:** Dextrose content repeatability, intermediate precision and accuracy.

Samples	Repeatability (% RSD Within-Day)	Intermediate Precision (% RSD Between-Day)	Accuracy (Bias in %)
Dextrose 1.44 mg/mL	1.48%	2.15%	0.05%
Dextrose 1.60 mg/mL	2.18%	1.84%	0.28%
Dextrose 1.76 mg/mL	1.50%	1.57%	−0.45%

RSD—relative standard deviation; n = 18.

**Table 2:** 5-HMF content repeatability, intermediate precision and accuracy.

Samples	Repeatability (% RSD Within-Day)	Intermediate Precision (% RSD Between-Day)	Accuracy (Bias in %)
5-HMF 10 µmol/L	0.913%	0.723%	0.333%
5-HMF 15 µmol/L	0.516%	0.448%	−0.063%
5-HMF 20 µmol/L	0.128%	0.667%	0.370%

RSD—relative standard deviation; 5-HMF—5-Hydroxymethylfurfural; n = 18.

**Table 3:** Forced dextrose degradation study.

Condition	Dextrose Degradation	5-HMF	Other Unidentified Compounds (RRT)
HCl 0.5 N, 0.5 h	19.7%	<LOD	0.31; 0.52; 0.58; 2.44
NaOH 0.5 M, 5 h	20.4%	<LOD	0.43; 0.52; 0.58; 0.68; 2.44
80 °C, 24 h	20.2%	58.6 µmol/L (7.40 µg/mL)	0.52; 0.68; 1.03; 1.10
Light, 32 days	18.2%	<LOD	0.52; 0.58; 0.77; 1.10; 2.44
KMnO_4_ 20 mg/mL, 2 h	20.7%	512.4 µmol/L (64.6 µg/mL)	0.24–0.81; 2.44

RRT—relative retention times; the retention times of other unidentified compounds were expressed relatively to the retention time of 5-HMF (11.2–11.6 min); LOD—limit of detection; 5-HMF—5-Hydroxymethylfurfural; n = 3.

**Table 4:** Stability of 40% (*w*/*v*) dextrose gel under refrigerated conditions (2–8 °C).

Parameter	DO	D2	D7	D15	D21	D30	D45	D60	D90
Dextrose (%) ^a^	40.4 ± 0.90	41.0 ± 0.41	40.7 ± 0.12	40.1 ± 0.22	40.0 ± 0.37	40.0 ± 0.72	39.4 ± 0.18	39.0 ± 0.58	41.1 ± 0.28
5-HMF	ND	ND	ND	ND	ND	ND	ND	ND	ND
pH ^a^	4.85 ± 0.01	4.84 ± 0.02	4.82 ± 0.02	4.78 ± 0.02	4.76 ± 0.02	4.80 ± 0.01	4.72 ± 0.03	4.80 ± 0.03	4.73 ± 0.03
Microbiological contamination	<LOD	<LOD	<LOD	<LOD	<LOD	<LOD	<LOD	<LOD	<LOD
Preservative efficacy	Efficient	NT	NT	NT	NT	Efficient	NT	Efficient	Efficient
Viscosity (mPa.s) ^a^	481.5 ± 19.3	484.9 ± 6.0	484.7 ± 14.9	501.6 ± 2.2	484.7 ± 4.7	492.1 ± 3.7	490.5 ± 2.2	486.6 ± 8.6	485.5 ± 9.7

^a^ Mean value ± standard deviation; 5-HMF—5-Hydroxymethylfurfural; LOD—limit of detection, 10 CFU/mL; ND—not detected; NT—not tested; Efficient—2 weeks after inoculation on agars, the logarithmic decrease in the microorganism count was, respectively, at least equal to a factor of 3 and 1 for the bacterial and fungal strains, and 4 weeks after inoculation, these values were lower than or equal to those reported 2 weeks after inoculation; n = 3.

**Table 5:** Stability of 40% (*w*/*v*) dextrose gel under ambient conditions (25 °C/60% RH).

Parameter	DO	D2	D7
Dextrose (%) ^a^	40.3 ± 0.90	41.1 ± 1.23	40.6 ± 0.19
5-HMF	ND	ND	ND
pH ^a^	4.84 ± 0.01	4.83 ± 0.02	4.81 ± 0.01
Microbiological contamination	<LOD	<LOD	<LOD
Preservative efficacy	Efficient	Efficient	Efficient
Viscosity (mPa.s) ^a^	481.5 ± 19.3	496.2 ± 6.4	497.7 ± 4.3

^a^ Mean value ± standard deviation; LOD—limit of detection, 10 CFU/mL; ND—not detected; Efficient—2 weeks after inoculation on agars, the logarithmic decrease in the microorganism count was, respectively, at least equal to a factor of 3 and 1 for the bacterial and fungal strains, and 4 weeks after inoculation, these values were lower or equal to those reported 2 weeks after inoculation; n = 3.

**Table 6:** Cumulative amounts of dextrose, caffeine and paracetamol that permeated per unit area versus time through artificial membrane impregnated with lipids.

Time (Hours)	Cumulative Amount of Dextrose (µg/cm^2^) ^a^	Cumulative Amount of Caffeine (µg/cm^2^) ^a^	Cumulative Amount of Paracetamol (µg/cm^2^) ^a^
0	0	0	0
0.5	0	272 ± 47	791 ± 43
1	0	791 ± 105	1464 ± 56
1.5	0	1294 ± 134	2208 ± 55
2	0	1948 ± 205	2809 ± 80
2.5	0	2470 ± 220	3353 ± 112
3	0	2917 ± 224	3916 ± 95
3.5	0	3586 ± 232	4317 ± 63
4	319 ± 63	4204 ± 131	4605 ± 324
6	645 ± 56	5660 ± 227	5048 ± 323
8	749± 110	6582 ± 539	6235 ± 326
10	922 ± 140	7020 ± 555	6808 ± 451

^a^ Mean value ± standard deviation; n = 8.

## Data Availability

Data are contained within the article.
